# Comparison of pre- and post-contrast myocardial T1 with histology findings in Experimental autoimmune myocarditis in rats

**DOI:** 10.1186/1532-429X-17-S1-W15

**Published:** 2015-02-03

**Authors:** Sarah Jeuthe, Katharina Wassilew, Darach O h-Ici, Frédéric H Münch, Hergen Payne, Patrick Schmerler, Felix Berger, Titus Kuehne, Daniel Messroghli

**Affiliations:** 1 German Heart Institute Berlin, Berlin, Germany; 2 Center for Cardiovascular Research, Berlin, Germany

## Background

Magnetic resonance imaging as a noninvasive method offers diagnostic and prognostic information in myocarditis. T1 mapping techniques aim to overcome the limitations of late gadolinium enhancement to assess diffuse fibrosis and inflammatory infiltrates. Using an established animal model of myocarditis, the aim of this study was to measure myocardial T1 before the onset, and in the acute and chronic stages of the disease and to compare its course with histological and immunohistochemistry findings.

## Methods

Male young Lewis rats were immunized with 0.25 mg porcine myocardial myosin into the rear footpads on day 0. Native and contrast-enhanced ECG-triggered cardiac MRI examinations were performed before immunization on day 0 and on days 14, 21 and 35 under isoflurane anesthesia. Left ventricular function and pre- and post- contrast T1 parameters were assessed using Small animal look-locker inversion recovery (SALLI). For each of the indicated time points a minimum of 4 rats were randomly investigated using conventional histology (H&E), histochemistry (Sirius-Red) and immunohistochemistry (CD68).

## Results

All immunized rats developed myocarditis (morbidity 100 %). Histologically we observed increased wall thickness with biventricular macrophage-rich mixed inflammatory infiltrates. All rats with histologically severe myocarditis showed increased native T1 and decreased post-contrast T1 of the myocardium. During disease progression, inflammatory infiltrates, fibrosis and native T1 increased until day 21, while post-contrast T1 decreased. In the chronic state of the disease (day 35), inflammatory infiltrates, collagen and native T1 decreased and post contrast T1 increased.

## Conclusions

The assessment of native T1 and post-contrast T1 allows for reliably differentiating between healthy myocardium and inflammatory infiltrated myocardium and between the acute and chronic stages of the disease.

## Funding

Sarah Jeuthe, this work is supported by a research grant of the German Cardiac Society (DGK).

